# Rehabilitation alone after anterior cruciate ligament injury yields greater limb symmetry but lower knee related self‐efficacy without limiting return to preinjury activity level

**DOI:** 10.1002/ksa.70042

**Published:** 2025-08-29

**Authors:** Rebecca Hamrin Senorski, Ramana Piussi, Johan Högberg, Roland Thomeé, Kristian Samuelsson, Eric Hamrin Senorski

**Affiliations:** ^1^ Sahlgrenska Sports Medicine Center Gothenburg Sweden; ^2^ Unit of Physiotherapy, Department of Health and Rehabilitation, Institute of Neuroscience and Physiology, Sahlgrenska Academy University of Gothenburg Gothenburg Sweden; ^3^ Department of Orthopaedics, Institute of Clinical Sciences, Sahlgrenska Academy University of Gothenburg Gothenburg Sweden; ^4^ Department of Orthopedics Sahlgrenska University Hospital Mölndal Sweden

**Keywords:** ACL reconstruction, anterior cruciate ligament, nonoperative, PRO, project ACL, rehabilitation

## Abstract

**Purpose:**

To compare patients treated with rehabilitation alone to those undergoing anterior cruciate ligament (ACL) reconstruction and rehabilitation with regard to recovery of muscle strength, return to knee‐strenuous sport and patient‐reported outcomes during the first 12 months of treatment.

**Methods:**

This study is a prospective cohort study, based on data from a rehabilitation registry, Project ACL, Gothenburg, Sweden. Included patients were ≥15 years with a primary ACL injury and had completed four follow‐ups under first year of treatment. Patients were divided into two groups, depending on treatment choice (1) rehabilitation alone (rehabilitation group), or (2) rehabilitation with ACL reconstruction (ACLR group). Absolute and symmetrical isokinetic muscle strength and patient reported outcomes were assessed using a predefined schedule. Analyses were adjusted for age at time of injury. Subanalyses were performed separately on muscle strength for female and male patients. Clinical relevance was assessed with Cohen's *d*.

**Results:**

In total, 31 patients in the rehabilitation group and 359 patients in the ACLR group were included. The rehabilitation group reported significantly greater symmetrical strength at every follow‐up, except 12‐month follow‐up for knee flexion, and better quality of life at 8 months. Female patients in the rehabilitation group were stronger in their injured limbs knee extension at 10‐week, 4‐month and 8‐month follow‐up as well as for the knee flexion at 10 weeks although no clinically relevant results. The ACLR group demonstrated significantly larger changes in limb symmetry from the 2‐ to 12‐month follow‐up, greater future knee self‐efficacy at 10 weeks, 4‐ and 8‐month follow‐up, and higher level of physical activity compared with the rehabilitation group, both preinjury, and at the 8‐ and 12‐month follow‐ups.

**Conclusion:**

Patients treated with rehabilitation alone recovered greater limb symmetry, while patients treated with ACLR had greater change in limb symmetry between 2 and 12 months after treatment. Patients in the ACLR group had higher future knee‐related self‐efficacy and were active at a higher level of physical activity at 8 and 12 months after treatment.

**Level of Evidence:**

Level II.

**Abbreviations**
ACL: anterior cruciate ligament
ACLR: anterior cruciate ligament reconstruction
ACL‐RSI: anterior cruciate ligament return to sport after injury scale
CI: confidence interval
cm: centimeters
d: cohen's *d*
ICC: interclass correlation coefficient
kg: kilogram
KOOS: knee injury and osteoarthritis outcome score
K‐SES: knee self‐efficacy score
LSI: limb symmetry index
M: months
n: number
nm: Newton metres
n/a: not applicable
PROs: patient reported outcomes
QoL: quality of life
RCT: randomised controlled trials
RECORD: REporting of studies Conducted using Observational Routinely‐collected Data
SD: standard deviation
STROBE: strengthening the reporting of observational studies in epidemiology
W: weeks
Δ: delta

## INTRODUCTION

Approximately 50% of patients who suffer an anterior cruciate ligament (ACL) rupture are treated with rehabilitation alone in Scandinavia [19]. Rehabilitative treatment can be associated with residual laxity of the knee joint and lower levels of physical activity after ACL injury [24, 25]. Rehabilitation alone may, however, result in less advanced radiographic knee osteoarthritis 10 years after an ACL injury [25], and less knee pain up to 21 years after the injury, compared with patients treated with ACL reconstruction (ACLR) [25].

Few randomised controlled trials (RCTs) have investigated the outcomes after ACL injury in patients treated with ACLR compared with rehabilitation alone [1, 4, 17, 30]. One study showed no differences in the knee injury and osteoarthritis outcome score (KOOS), including KOOS_4_, or the Tegner activity scale, between treatment groups [17] while another showed greater improvements for patients treated with ACL reconstruction [4]. Furthermore, patients treated with ACLR have showed a significant difference in the International Knee Documentation Committee scores at 3 months and at 2 years after treatment [30]. Collectively, the results from previous RCTs present both ACLR and rehabilitation alone as viable treatment options [1, 4, 17, 30]. However, 38% [17], 41% [4] and 50% [30] of the patients randomised to rehabilitation alone, opted for delayed reconstruction, which suggests that not all patients cope with this treatment.

Beyond RCTs, registry data suggest that rehabilitation alone results in inferior subjective knee function, most prominent in the KOOS subscales of function in sports and recreation and quality of life [2, 8, 27]. Since previous studies that compared outcomes after rehabilitation alone or ACLR generally focused on patient‐reported outcomes (PROs) [4, 8, 17, 27, 30], data on objective outcomes such as results of muscle strength tests are scarce. There is a need to improve our understanding of how patients' treatment choices may influence muscle strength recovery, activity level and perceived knee function during the first 12 months after treatment for an ACL injury.

The purpose of this study was to compare patients treated with rehabilitation alone with patients treated with ACLR plus rehabilitation, in terms of muscle strength, return to knee‐strenuous sport and perceived knee function during the first 12 months of treatment.

## MATERIALS AND METHODS

This study used the REporting of studies Conducted using Observational Routinely‐collected Data (RECORD), an extended version of Strengthening the Reporting of Observational Studies in Epidemiology (STROBE), as guidance [7]. The present study was designed as a prospective cohort study, based on data from a Swedish rehabilitation outcome registry, Project ACL. Project ACL aims to improve care for patients with an ACL injury irrespective of treatment [20]. Participation is voluntary and written consent is acquired prior to inclusion. Patients are invited to undergo an evaluation with PROs and muscle function tests according to a predefined schedule, at 10 weeks and 4, 8, 12, 18 and 24 months, followed by every fifth year after baseline (injury/reconstruction). The study was conducted in accordance with the Declaration of Helsinki [38].

### Test protocol

In Project ACL, the test of muscle function consists of unilateral isokinetic muscle strength tests for the quadriceps and hamstring muscles (knee extension and flexion) and hop tests, which include the vertical hop, hop for distance, and the 30‐s side‐hop. All muscle function tests are supervised by trained physiotherapists. At the time of the muscle function tests, patients are instructed to warm up according to a standardised procedure, previously published [28]. Unilateral muscle strength tests are performed in a seated isokinetic dynamometer at an angular velocity of 90° per second (Biodex System 4; Biodex Medical System). The Biodex dynamometer is considered reliable to test muscle strength, with an interclass correlation coefficient (ICC) of 0.95 [16]. Three to four single repetition maximum trials with 40 s of rest between each set are executed, and maximum torque in Newton‐metres (Nm) is registered in Project ACL's database and used for analysis. In the present study, only muscle strength tests were analysed.

Within 2 weeks of the muscle function tests, patients are invited to answer PROs digitally through a web‐based platform. The PROs include the KOOS (except for the activities of daily living subscale), the knee self‐efficacy scale (K‐SES) and the ACL return to sport after injury scale (ACL‐RSI), all of which were assessed in this study (Table 1).

**Table 1:** Patient‐reported outcomes, aims, items, subscales and scoring range for the outcomes used in this study.

PRO	Aim	Items	Subscales	Scoring range	Reliability	Content validity
KOOS [31]	Assess self‐reported symptoms following a knee injury	42	Pain Symptoms Activities of daily living (not used in this study) Sports and recreation Quality of life	0–100 Severe symptoms—no symptoms	ICC = 0.85–0.9 [13]	No evidence [18]
K‐SES_18_ [5]	Measure perceived knee‐related self‐efficacy in patients with an ACL injury	18	Present knee self‐efficacy Future knee self‐efficacy	0–10 for each item Poor—strong self‐efficacy	ICC = 0.92 [5] Cronbach's *α* = 0.81–0.96	Fair/positive [18]
ACL‐RSI [36]	Measure confidence, emotion and risk appraisal towards RTS after an ACL injury	12	n/a	0–10 for each item extremely negative—extremely positive	Cronbach's *α* = 0.92 [36]	No evidence—Fair/positive [18]
Tegner activity scale [35]	Assess the level of knee‐strenuous activity	1	n/a	1–10 Walking on even ground—elite sports. Level 6 represents the lowest level of sports participation.	ICC = 0.9 [35]	Acceptable floor and ceiling effects [10]

Abbreviations: ACL‐RSI, anterior cruciate ligament‐return to sport after injury scale; ICC, interclass correlation coefficient; KOOS, knee injury and osteoarthritis outcome score; K‐SES, knee self‐efficacy score; n/a, not applicable; PRO, patient‐reported outcome.

### Patients

Data were extracted from Project ACLs database on 2 May 2024. Eligible patients were those registered in Project ACL, with a primary ACL injury treated with rehabilitation alone or with ACLR within 12 months from injury and subsequent rehabilitation and, had data from all follow‐ups of muscle function and PROs within the first 12 months after injury/reconstruction. To allow for the 12‐month follow‐up of muscle strength and PROs, patients registered after December 2022 were not considered eligible for inclusion. Patients who were <15 years of age at time of injury or reconstruction, had not registered their age, had sustained a subsequent ACL injury within 12 months from baseline, had an unknown time from injury to surgery, had surgery within 12 months from injury on contralateral side, or chose to cross over from ACL rehabilitation to reconstruction within 12 months from injury were excluded.

### Groups

The included patients were allocated to two treatment groups. One group consisted of patients treated with ACL rehabilitation alone (rehabilitation group), and the other group consisted of patients who had undergone ACLR within 12 months from injury, followed by rehabilitation (ACLR group).

### Outcomes

Outcomes at all Project ACL's follow‐ups, that is, 10 weeks, 4, 8 and 12 months after ACL injury/reconstruction, were compared between treatment groups. The primary outcome was the recovery of symmetrical muscle strength. Recovery was defined as a limb symmetry index (LSI) ≥90% that is, result from the injured leg/results from the noninjured leg × 100.

Secondary outcomes of interest were absolute muscle strength values relative to body weight (Nm/kg), answer to PROs as well as return to sports, that is, measured with Tegner, where return to sport was defined as return to Tegner ≥6, at each of Project ACL's follow‐up assessments used in the study. The results of muscle strength tests were reported both as LSI and as Nm/kg.

### Statistical analysis

Statistical analysis was performed using SAS Statistics for Windows, Version 9.4 (SAS Institute Inc.). A priori sample size calculation was made with expected 10% difference between groups for the main outcome of quadriceps LSI with 80% power and an alpha of 0.05, suggesting that 16 patients would be required in each group. Means were presented with standard deviation (SD) and 95% confidence interval (CI) for all parametric variables. Where appropriate, medians were presented with minimum to maximum and interquartile range. Categorical variables were reported as frequencies (*n*) and proportions (%). Comparisons were made for each outcome with Fisher's exact test for dichotomous variables, Fisher's nonparametric permutation test for continuous variables and the Mantel‐Haenszel chi‐square test for ordered categorical variables. If no exact limit could be computed, the asymptotic Wald confidence limit with continuity correction was calculated instead. All analyses were adjusted for age at injury using logistic regression, as younger patients are more often advised treatment with ACL reconstruction [14]. Absolute muscle strength values were analysed for males and females separately. The CI for the mean difference between groups was based on Fisher's nonparametric permutation test. The alpha level was set at ˂0.05. Difference between groups was presented as delta (Δ). To interpret the clinical relevance of significant findings, effect sizes was calculated for numerical variables and Cohen's *d* was used with the following reference values: ≥0.20 = small, ≥0.50 = medium and ≥0.80 = large [34].

## RESULTS

**Figure 1:**
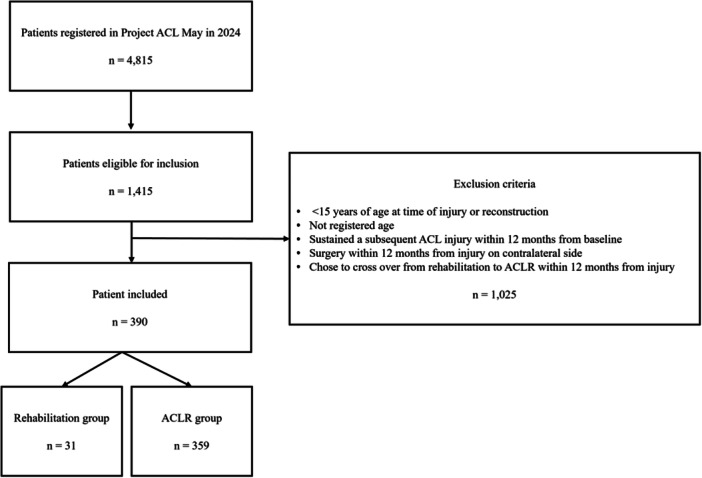
Flow chart of the inclusion process. ACL, anterior cruciate ligament; ACLR, anterior cruciate ligament reconstruction; *n*, number.

Figure 1 presents the selection process. A total of 390 patients had complete follow‐ups from baseline to 12 months after treatment, of which 31 patients were included in the rehabilitation group and 359 patients in the ACLR group.

Table 2 presents patient demographics stratified by treatment group. Patients in the rehabilitation group were significantly older at time of injury compared to patients in the ACLR group (*p* < 0.001). Patient sex, height or weight did not differ between groups.

**Table 2:** Demographics for included patients stratified by treatment group.

	Rehabilitation group (*n* = 31)	ACLR group (*n* = 359)	*p*‐Value	Δ (95% CI)	Effect size
Age at injury mean ± SD	41.3 ± 10.3	26.0 ± 10.4	<0.001 <0.001	17.1 (13.2; 20.8)	1.64
Male	45.2 ± 7.6	25.2 ± 10.5		19.9 (14.8; 24.5)	1.93
Female	39.8 ± 13.2	27.0 ± 10.3		10.9 (6.8; 14.7)	1.13
Sex, *n* (%)					
Male	19 (61.3)	210 (58.5)	0.92	2.8 (−16.8; 22.4)	0.06
Female	12 (38.7)	149 (41.5)			
Height, cm mean ± SD					
Male	180 ± 8	181 ± 7	0.43	−1.6 (−5.6; 2.3)	0.24
Female	167 ± 5	169 ± 6	0.30	−1.5 (−4.2; 1.3)	0.25
Weight, kg mean ± SD					
Male	78.1 ± 10.7	80.9 ± 10.1	0.36	−2.9 (−9.0; 3.0)	0.28
Female	66.2 ± 10.7	66.5 ± 8.6	0.90	−0.4 (−4.7; 3.6)	0.04

*Note*: Effect size was calculated with Cohen's *d*.

Abbreviations: ACL, anterior cruciate ligament; ACLR, anterior cruciate ligament reconstruction; CI, confidence interval; n, number; SD, standard deviation.

### Muscle strength

**Figure 2:**
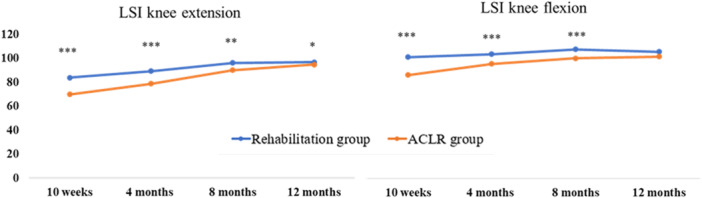
Development of knee extension and flexion limb symmetry index throughout the first 12 months after ACL injury or reconstruction for the respective treatment group. *p < 0.05, **p < 0.01, ***p < 0.001. ACL, anterior cruciate ligament; CI, confidence interval; LSI, limb symmetry index; Δ, difference between groups (delta).

All analyses were adjusted for age at injury using logistic regression. Patients treated with rehabilitation alone demonstrated significantly greater LSI for knee extension strength at 10 weeks (*p* < 0.001, *d* = 0.93), 4 months (*p* = 0.0002, *d* = 0.68), 8 months (*p* = 0.0013, *d* = 0.48) and 12 months (*p* = 0.035, *d* = 0.196), respectively, compared with treatment with ACLR (Figure 2). The rehabilitation group had greater LSI for knee flexion strength at the 10‐week (*p* < 0.001, *d* = 1.04), 4 months (*p* = 0.0006, *d* = 0.64) and 8 months (*p* = 0.007, *d* = 0.67) follow‐up compared to the ACLR group.

**Figure 3:**
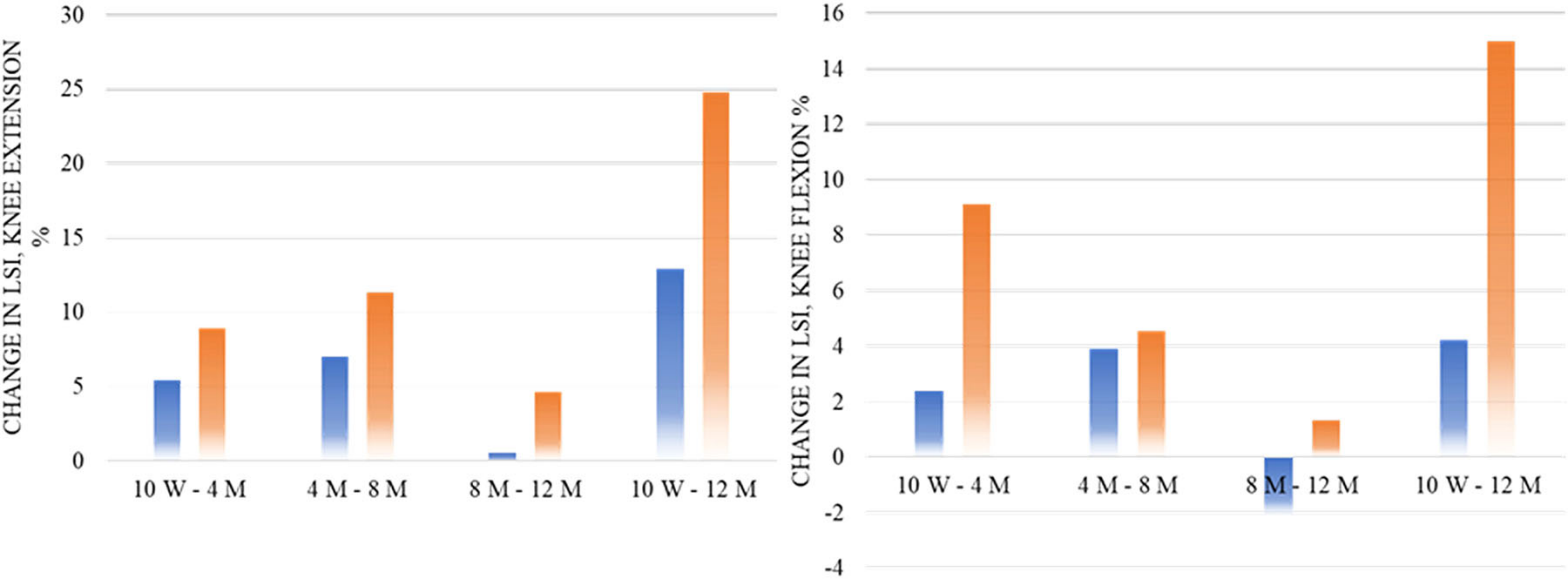
Differences in the limb symmetry index between groups and follow‐ups throughout the first 12 months after ACL injury or reconstruction for the respective treatment groups. *p < 0.05, **p < 0.01, ***p < 0.001. ACL, anterior cruciate ligament; CI, confidence interval; LSI, limb symmetry index; M, months; W, weeks.

Patients in the rehabilitation group demonstrated inferior change in knee extension LSI compared to the ACLR group, between the 4‐ and 8‐month follow‐up, 7.0% ± 9.9% versus 11.3% ± 9.2% (Δ −4.33, 95% CI −7.90; −0.78, *p* = 0.018) and between 10 weeks and 12 months 12.9% ± 14.9% versus 24.8% ± 12.4%, (Δ −11.9, 95% CI −16.7; −7.0, *p* = 0.0002) (Figure 3).

Patients treated with rehabilitation alone also displayed inferior change in knee flexion LSI compared to treatment with ACLR between follow‐ups 10 weeks and 4 months 2.4% ± 10.4% versus 9.1% ± 10.9% (Δ −6.68, 95% CI −10.93; −2.64, *p* = 0.0017), and between 10 weeks and 12 months 4.2% ± 14.2% versus 15.0% ± 13.0% (Δ −10.8, 95% CI −15.9; −5.9, *p* < 0.001).

### Comparison between sexes

**Figure 4:**
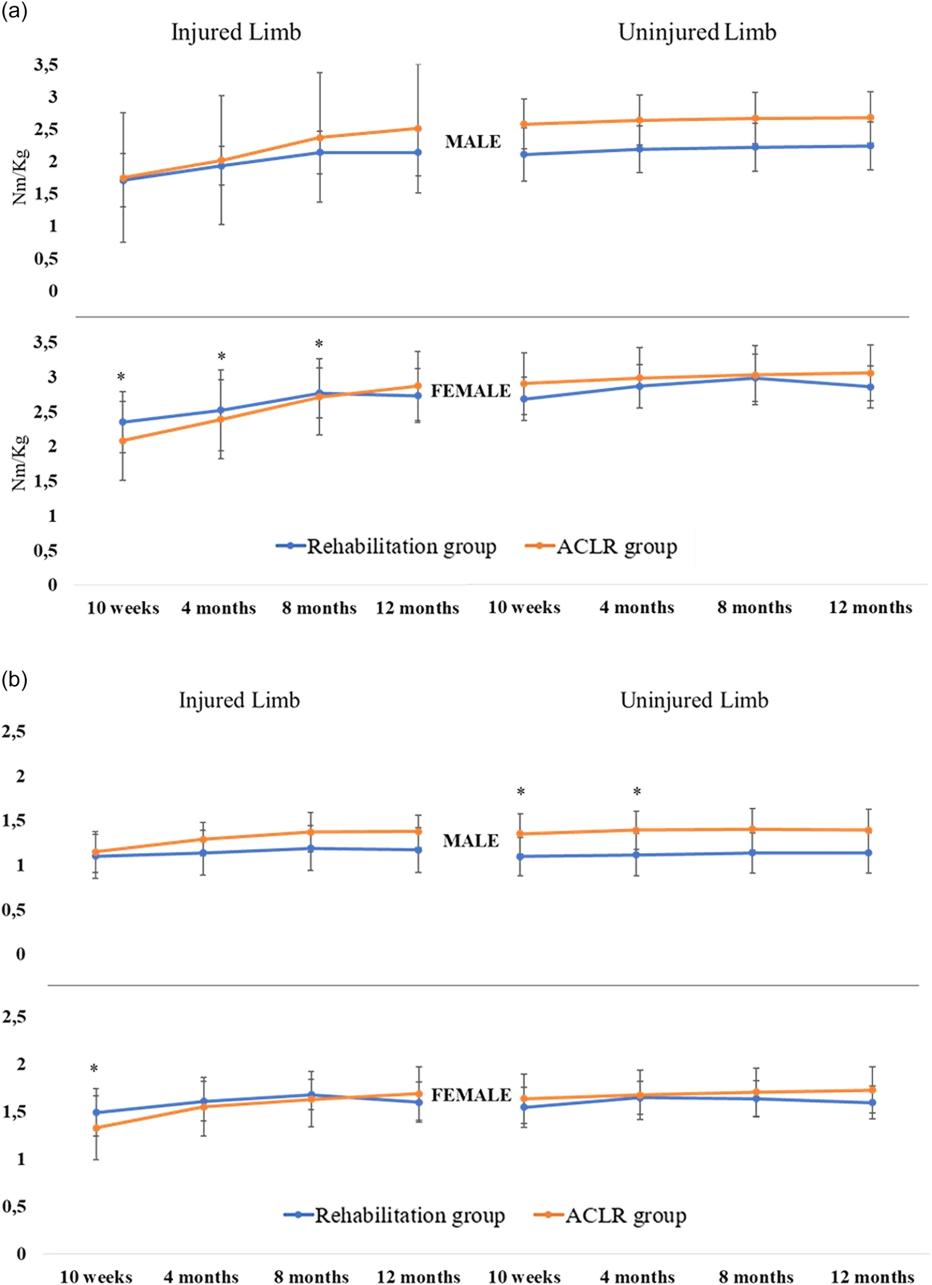
(a) Comparisons between treatment groups for absolute strength for the knee extension, expressed in Nm in relation to body weight. Graphs are stratified by sex, as well as injured versus uninjured limb. *p < 0.05, **p < 0.01, ***p < 0.001. (b) Comparisons between treatment groups for absolute strength in the knee flexion, expressed in Nm in relation to body weight. Graphs are stratified by sex, as well as injured versus uninjured limb. *p < 0.05, **p < 0.01, ***p < 0.001. ACL, anterior cruciate ligament; Nm/kg, Newton metres per kilogram body weight.

Females treated with rehabilitation alone were stronger in their injured limbs knee extension in Nm/kg compared to females treated with ACLR at 10 weeks 2.35 ± 0.44 versus 2.08 ± 0.57 (Δ 0.27, 95% CI −0.06; 0.60, *p* = 0.01, *d* = 0.49), at 4 months 2.52 ± 0.58 versus 2.39 ± 0.57 (Δ 0.12, 95% CI −0.21; 0.47, *p* = 0.02, *d* = 0.22), and 8 months 2.77 ± 0.36 versus 2.71 ± 0.55 (Δ 0.06, 95% CI −0.26; 0.37, *p* = 0.02, *d* = 0.10) (Figure 4a). Females treated with rehabilitation alone were also stronger in the injured limbs knee flexion at the 10‐week follow‐up 1.49 ± 0.25 versus 1.33 ± 0.34 (Δ 0.16, 95% CI −0.05; 0.35, *p* = 0.032, *d* = 0.47) (Figure 4b).

Males in the rehabilitation group were weaker in their uninjured limbs knee flexion strength compared to males in the ACLR group at 10 weeks: 1.10 ± 0.22 versus 1.35 ± 0.21 (Δ −0.25, 95% CI −0.35; 0.15, *p* = 0.012, *d* = 1.17) and at 4 months 1.12 ± 0.21 versus 1.39 ± 0.23 (Δ −0.26, 95% CI −0.37; 0.16, *p* = 0.046, *d* = 1.15) (Figure 4b). There was no significant difference in knee extension strength for males independent of limb or treatment.

### PROs

In terms of the KOOS subscales, the rehabilitation group reported higher QoL than the ACLR group, 63.5 ± 15.0 versus 56.4 ± 17.1 (Δ 7.0, 95% CI 0.7; 13.2, *p* = 0.03) at 8 months. There were no other differences in the other KOOS subscales between treatment groups throughout the study follow‐ups (Appendix Figure S1a and S1b).

Patients in the rehabilitation group had lower future knee‐related self‐efficacy than the ACLR group, at 10 weeks 6.5 ± 1.8 versus 7.5 ± 1.6 (Δ −1.1 95% CI −1.6; 0.4, *p* = 0.001), 4 months 6.8 ± 1.9 versus 7.6 ± 1.6 (Δ −0.8 95% CI −1.4; 0.2, *p* = 0.01), and at 8 months 7.7 ± 1.6 versus 7.0 ± 2.2 (Δ −0.7 95% CI −1.2; 0.03, *p* = 0.04), (Appendix Figure S2), respectively. No differences were observed between groups for the K‐SES_present_ at any of the follow‐ups (Appendix Figure S2), or for the ACL‐RSI at the 8‐ and 12‐month follow‐up (Appendix Figure S3).

**Figure 5:**
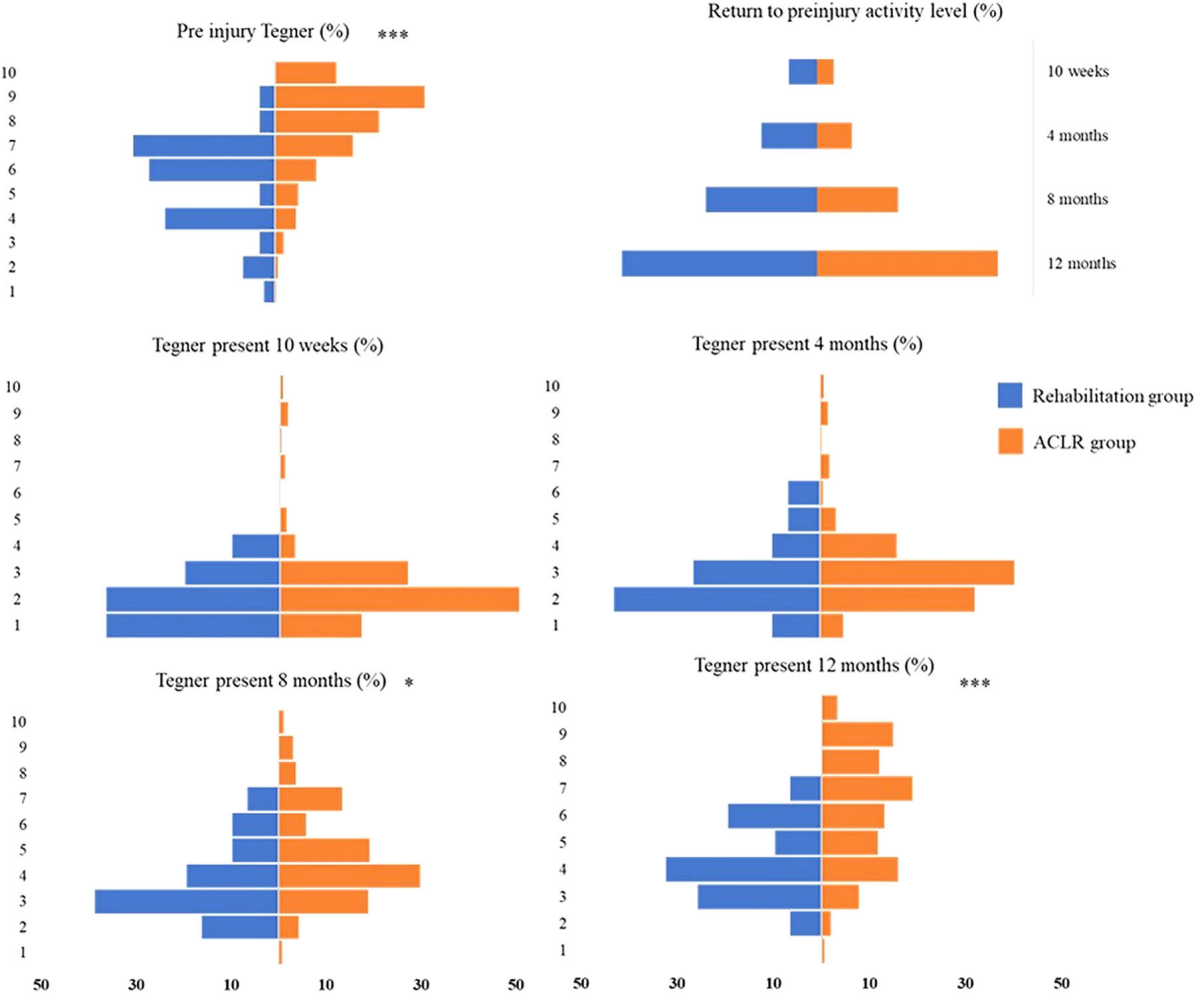
The Tegner activity scale for up to 12 months after anterior cruciate ligament (ACL) injury or reconstruction, the *Y*‐axis 1–10 represents the different Tegner activity levels. *p < 0.05, **p < 0.01, ***p < 0.001. The blue bars denote the rehabilitation alone group, while the orange bars denote the ACLR group.

A larger proportion of patients treated with ACLR were active at a higher level of knee‐strenuous activity, measured with the Tegner activity scale, both preinjury (*p* < 0.001), at 8 months (*p* = 0.003) and at the 12‐month follow‐up (*p* < 0.001). There was no significant difference between treatment groups in the proportions of patients who returned to their preinjury activity level at any follow‐up. At 12 months after treatment, 38.7% had returned to preinjury activity level in the rehabilitation group versus 36.8% in the ACLR group (Figure 5).

## DISCUSSION

The most important findings of our study were that patients treated with rehabilitation alone had greater knee extension strength LSI for all follow‐ups and greater knee flexion strength LSI at 2, 4 and 8 months, however, lower change in LSI over time and inferior future knee‐related self‐efficacy group up to the 12‐month follow‐up compared with patients in the ACLR group. While male patients in the ACLR group were significantly stronger in knee flexion in their uninjured limbs at 10‐week and 4‐month follow‐up, females in the rehabilitation group displayed stronger knee extension in the injured limb at 2‐, 4‐ and 8‐month follow‐up and stronger injured limbs knee flexion at 2 month follow‐up. Only the differences in knee flexion strength between male patients in the two treatment groups were considered clinically relevant.

### Muscle strength tests

To achieve an LSI of ≥90% in knee extension and flexion strength at the end of rehabilitation after ACL injury/reconstruction is regarded as part of achieving a successful outcome [26], although it has previously been reported that limb symmetry can in some cases be achieved through bilateral weakness [37]. In the present study, patients treated with rehabilitation alone displayed greater limb symmetry compared to patients treated with ACLR. However, patients treated with ACLR have a greater increase in limb symmetry between 10 weeks and 12 months. In line with previous research, patients treated with ACLR display greater limb asymmetries early in their rehabilitation, but differences are reduced over time [15]. Treatment with ACLR induces major muscular atrophy in the quadriceps muscle, which may partly explain the greater asymmetries in knee extension at 10 weeks for the ACLR group which are later in rehabilitation recovered. The increase in limb symmetry may be a result of greater recovery in patients treated with ACLR due to the early muscle atrophy that is not as present in patients treated with rehabilitation alone.

Previous research has suggested that patients treated with ACLR can struggle to regain their preoperative absolute strength, despite regained LSI in muscle strength tests [29]. In the present study, females treated with rehabilitation alone showed significant greater absolute knee extension strength in their injured limb compared to the ACLR group, however, males presented greater knee flexion strength in their contralateral limb. A weakness in the contralateral limb has previously been reported in patients treated with ACLR for isometric quadriceps and eccentric knee flexion strength [15]. Results from the present study assessed with effect sizes between rehabilitation and ACLR groups were only clinically relevant for knee flexion strength in the uninjured limb in male patients. A stronger knee flexion in the uninjured limb in early stages of rehabilitation (10 weeks and 4 months) could partly be explained by the harvest of the ACL graft which induces weaknesses in the injured limb. Furthermore, the contralateral weakness in knee flexion may be due to limited studies on rehabilitation guidelines for patients treated with specific semitendinosus autografts [9]. However, the choice of autograft was not considered in the present study.

In summary for results of muscle function tests, clinicians can expect patients treated with rehabilitation alone to regain limb symmetry earlier in the rehabilitation process compared to patients treated with ACLR.

### PROs

In the present study, the ACLR group scored higher on the KOOS subscale quality of life at 8 months, which confirms previously published results [2]. However, the difference in our study was not considered clinically relevant as it did not exceed the minimal clinically important difference [6]. A cross‐sectional comparative study [2] reported that patients treated with ACLR have significantly less knee pain and symptoms, as measured with the KOOS, at the 2‐year follow‐up compared with patients treated with rehabilitation alone. On the contrary, less knee pain has been reported for patients treated with rehabilitation alone after 15 and 20 years compared to patients treated with ACLR [25]. Results from our study suggest no significant differences in the KOOS pain or symptoms at any follow‐up, however, in comparison with previous studies, our follow‐up was at 12 months. In summary, patients with an ACL injury independent of treatment may display a change in symptoms and pain over time.

In the present study, patients treated with ACLR displayed significantly higher K‐SESfuture at 10 weeks, 4 and 8 months after baseline, compared to the rehabilitation group. Patients treated with rehabilitation alone have reported a lack of trust in their knee during activities [32], which might be a contributing reason that patients treated with rehabilitation alone lower their activity level after an ACL injury [23]. In combination with greater K‐SES future, patients in the ACLR group demonstrated greater knee flexion strength. Previous literature has reported significant but weak correlations between absolute strength and the K‐SES_18_ at multiple time points up to 12 months [21], which indicate that while strength may play a role in shaping self‐efficacy beliefs, particularly future function, other psychological factors such as confidence, emotion and risk appraisal, and contextual factors like sport participation are also likely to contribute to the knee related self‐efficacy. Patients with higher present knee self‐efficacy have previously been reported with greater satisfaction with rehabilitation outcomes following ACL injury [3] and less impairment following ACLR [12]. Patients treated with rehabilitation alone might thereby have a greater need for assistance to boost knee‐related self‐efficacy during the rehabilitation.

In our study, patients treated with ACLR reported a higher level of physical activity preinjury, at 8 months and at 12 months after reconstruction, but there was no difference in rates of return to preinjury activity level between treatment groups. In line with previous research, patients with higher preinjury activity levels are more often treated with ACLR compared with patients with lower activity levels [11, 14, 33], and only patients who are willing to modify their activity level should be recommended rehabilitation alone as treatment [23]. A lower preinjury activity level might lead to higher rates of return to preinjury activity levels. Consequently, patients treated with rehabilitation alone might display similar rates of return to preinjury level as patients treated with ACLR.

### Limitations

One limitation of the current study was there is a risk for confounding by indication, that is, there was no information on why patients received rehabilitation alone or ACLR as treatment. There were no details on the specific content in the patients rehabilitation in the registry, but there were no expected differences with regard to strength training, and all patients underwent the same evaluations with reports sent to treating physical therapists for guidance. Training volume was not presented for either of the treatment groups and was thus not accounted for.

Another limitation relates to the differences in baseline demographics between the groups, which limit the external validity of our results. Patients treated with ACLR were 14 years younger on average than patients treated with rehabilitation alone. In an attempt to account for age differences in the cohort, we adjusted all analyses for age at time of injury. Furthermore, the use of the Tegner activity scale only determines the level of knee‐demanding activity, which limits the results in regard to patients' participation in physical activity, as the Tegner does not account of the amount of physical activity, time of exposure, or intensity. These limitations reflect previous literature where younger patients who are more physically active preinjury are more likely to receive ACLR as primary treatment. In addition, there were no data on concomitant injuries such as associated meniscal or ligament injuries with may impact the choice of treatment and outcomes [22]. Neither tibial slope nor alignment was taken into consideration. Thus, results from the present study should be interpreted with caution.

We have performed several between‐group comparisons, and the results are therefore at risk of type‐I errors. In an attempt to address this, effect sizes were calculated for all between‐group analyses to assess if differences were clinically relevant. Taken together, the results of our study should be interpreted with caution and cannot be generalised to all patients treated with rehabilitation alone or ACLR after an ACL injury.

### CONCLUSION

Patients treated with rehabilitation alone recovered greater limb symmetry across all follow‐ups except for knee flexion at 12 months, while patients treated with ACLR had a greater change in limb symmetry between 2 and 12 months after treatment. In addition, patients treated with ACLR had higher future knee‐related self‐efficacy and were active at a higher level of physical activity at 8 and 12 months after treatment.

## AUTHOR CONTRIBUTIONS

Rebecca H. Senorski, Ramana Piussi, Roland Thomée, Kristian Samuelsson and Eric H. Senorski contributed to the concept and design of the study. Roland Thomée designed and organised the database together with Eric H. Senorski. Rebecca H. Senorski and Ramana Piussi performed the data analysis with the assistance of a statistician at Statistiska konsultgruppen and wrote a draft of the manuscript. Johan Högberg, Ramana Piussi and Kristian Samuelsson wrote sections of the manuscript. All the authors contributed to the manuscript revision and read and approved the submitted version.

## CONFLICTS OF INTEREST STATEMENT

Kristian Samuelsson reports a relationship with Getinge AB that includes: board membership. Eric H. Senorski is the associate editor of Journal of Orthopeadic and Sports Physical Therapy. The remaining authors declare no conflicts of interest.

## ETHICS STATEMENT

Ethical approval has been obtained from the Swedish Ethical Review Authority (registration number: 2024‐08724‐01, 2020‐02501) and the Regional Ethical Review Board in Gothenburg, Sweden (registration numbers: 265‐13, T023‐17).

## Supporting information


**Appendix Fig. 1a** Comparisons between treatment groups for KOOS Pain and Symptoms. *p<0.05, **p<0.01, ***p<0.001. ACL; Anterior Cruciate Ligament, CI; Confidence Interval, KOOS; Knee injury and Osteoarthritis Outcome Scale, Δ; Difference between groups delta.

**Appendix Fig. 1b** Comparisons between treatment groups for KOOS Sports and QoL. *p<0.05, **p<0.01, ***p<0.001. ACL; Anterior Cruciate Ligament, CI; Confidence Interval, KOOS; Knee injury and Osteoarthritis Outcome Scale, QoL; Quality of Life, Δ; Difference between groups delta.

**Appendix Fig. 2** Comparisons between treatment groups for K‐SES present and future. *p<0.05, **p<0.01, ***p<0.001. ACL; Anterior Cruciate Ligament, CI; Confidence Interval, K‐SES; Knee‐Self Efficacy Scale, Δ; Difference between groups delta.

**Appendix Fig. 3** Comparisons between treatment groups for ACL‐RSI. *p<0.05, **p<0.01, ***p<0.001. ACL; Anterior Cruciate Ligament, CI; Confidence Interval, K‐SES; Knee‐Self Efficacy Scale, Δ; Difference between groups delta.

## Data Availability

Data are available upon request.
