# Supplementary material for: Rehabilitation alone after anterior cruciate ligament injury yields greater limb symmetry but lower knee related self‐efficacy without limiting return to preinjury activity level
Source: Knee Surg Sports Traumatol Arthrosc. 2025 Aug 29;33(12):4435–45. doi: 10.1002/ksa.70042 (PMC12684339; doi:10.1002/ksa.70042)
Supplement: Supplementary file 3 — Appendix Fig. 2 Comparisons between treatment groups for K‐SES present and future. *p<0.05, **p<0.01, ***p<0.001. ACL; Anterior Cruciate Ligament, CI; Confidence Interval, K‐SES; Knee‐Self Efficacy Scale, Δ; Difference between groups delta. [file KSA-33-4435-s003.docx]

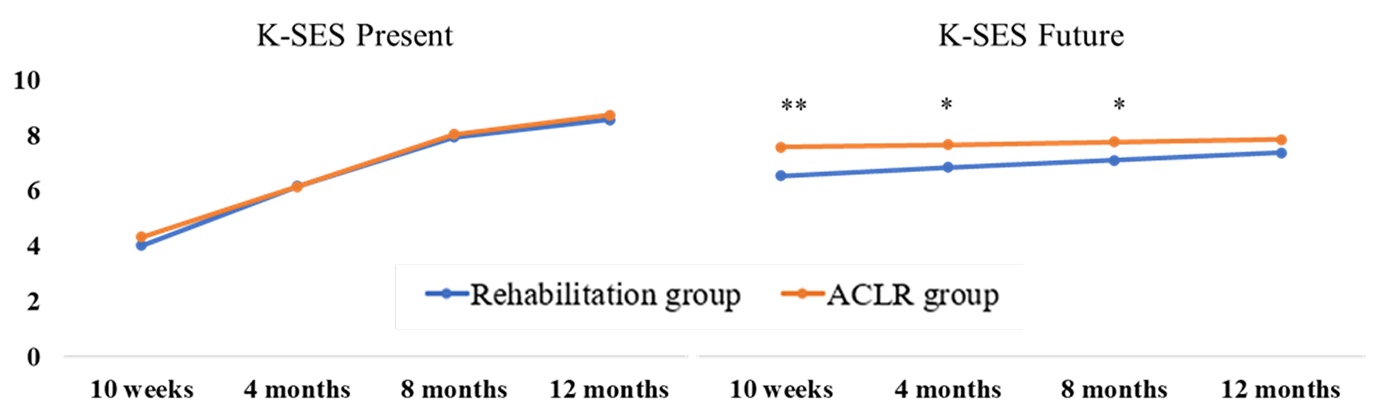


**Appendix Fig. 2** Comparisons between treatment groups for K-SES present and future. *p<0.05, **p<0.01, ***p<0.001. ACL; Anterior Cruciate Ligament, CI; Confidence Interval, K-SES; Knee-Self Efficacy Scale, Δ; Difference between groups delta.
